# Nurse Marion's Romance

**Published:** 1887-03-26

**Authors:** Kate Furley


### Nurse Marion's Romance.

By Kate Furley.

Chapter I.—A Surprising Proposal.

"This is fortunate!" exclaimed Dr. Brandreth, one of the physicians at St. Lazarus' Hospital, as his eye fell on the slim, graceful figure of a lady, whose sweet, refined face seemed the brighter by contrast with the plain black bonnet she wore. The rest of her dress consisted of a plain dark gown, and a long cloak made without any reference to the decrees of the prevailing fashion; but, in the eyes of Charles Brandreth, M.D., the wearer of these simple garments was more attractive than the most gaily dressed woman in the world.

She was walking slowly across the Park, the near proximity of which to St. Lazarus's was one of the characteristics of that hospital most valued by those who worked there, and seemed to be enjoying the fresh spring air and the sight of children playing on the grass, when the Doctor observed her.

He stopped his carriage, alighted, and having directed his coachman to drive on, joined the lady.

"I am so glad to have met you, Nurse Marion," he exclaimed. "I have just been at the hospital, forgetting that this was your off time. I wanted to see you particularly."

"Is there anything you want me to do?" asked the nurse.

"Yes, I have been asked to take in a patient who is now on his way home from India," said Dr. Brandreth. "I think we had better put him in the little private ward at the end of yours, as he is going to pay, and only comes to the hospital because he seems to have no relations to look after him. It's rather a dreary thing to have no one to whose care and affection one has a right, and our unknown patient is wise to come to a place where skill can in some degree atone for the absence of love. When I am dying I shall make my servants bring me to the very same little ward where we are going to put this stranger, and I will have you to tend me during my last hours. I could die very happily, I think, if you were near me, looking as divinely pitiful as you do when some of the rough specimens of humanity in the ward are crossing the dark river."

A slight contraction of Nurse Marion's delicate eyebrows showed that she would have preferred that the Doctor's flattering words had been left unuttered; and her reply was exclusively confined to the question of the impending invalid.

"What is your patient's name?" she asked, "and what is his disease?"

"As the telegraph clerks have worked their will on his message, I can only guess at the name. It seems to be Hatton, or something the sort. He says he is suffering from paralysis, which may mean anything between anæmia and apoplexy;—you can't take a patient's word about his ailments."

"Perhaps the climate of India has injured him."

"Climate plus brandy, probably. Half the diseases for which India gets blamed are due to self-indulgence, which would bring a man to death's door in any country."

"When will this Mr. Hatton arrive?"

"The day after to-morrow, if his vessel is up to time. You will have the ward ready?"

"Yes."

"And—and—Marion," said the Doctor, stammering in a way that seemed rather strange in a man of forty, who had the reputation of possessing as much *sang-froid* as anyone in England, and who had made for himself, by sheer talent and determination, one of the best positions in the medical world. His diagnosis was considered infallible, but he was now trying to diagnose a case which demanded knowledge not physiological but psychological, and feeling himself at fault, he stammered like a schoolboy.

"Have you anything further to say to me, Dr. Brandreth?" asked the nurse, with as much austerity in her tone as a voice attuned naturally only to tenderness could convey. "I ought to be at the hospital now, and I think it is nearly time for you to meet your class. You will have to hasten to the lecture-room."

She was about to walk away, but Dr. Brandreth laid his hand on hers, and detained her.

"Spare me a few more seconds," he said. "I have something to say to you which I cannot put off longer. Marion, you have been nurse in this ward of mine for nearly three years. I have seen you daily all that time; it is not strange that I should have grown to love you. Strange! Does not every patient who has been under your influence for a day or two worship the ground you tread on, the shadow you cast as you pass? They are coarse and hardened rascals, many of them, yet everyone yields to your sweetness; and it would be strange indeed if I escaped its power. To me you are simply all that I have dreamed of in women—good, and brave, and tender——"

"Stop, Dr. Brandreth!" cried Marion, blushing as hotly as if she had been a girl of sixteen instead of a woman of thirty, chastened by long experience of suffering, her own and others, till one might have thought her dead to violent emotion. "You must not talk so," she cried. "I don't deserve such praise; and even if I did, you should not——"

"I should, and must, and will!" he answered. "I must tell you all now, Marion; and I have a right to insist that you should listen to me when I tell you that I love you, and ask you to be my wife."

Nurse Marion grew pale now, and her voice trembled, while the Doctor suspected, though the long-fringed lids concealed them, that there were tears in her clear brown eyes. "It is impossible," she said sadly, "quite impossible."

"But why?"

She hesitated, and a flickering colour again mounted to her cheeks.

"You know what a prejudice exists against doctors marrying nurses."

"I know it, and I understand the cause of it. No one could object more strongly than I to a girl coming to a hospital as she might to a ball, with a view only to amusing herself, and catching a husband. I have frequently protested against this modern institution of lady probationers, nurses, or short-timers, for the very reason that those who are not compelled to earn their living by the work they have undertaken are apt to neglect it, and spend their time in flirting with students and house-surgeons. That is a thing which the authorities of every hospital must discourage, and put a stop to. But this is a different matter. If a man sees a woman who is all that he could picture his ideal to be, who is strong without being unfeminine, tender without being weak, intelligent and pure-souled and beautiful, is he to be forbidden to love her because she chances to be a nurse and he a doctor, and because he met her first in the wards of a hospital? I hold myself free to love and marry any woman whom I admire and respect, if she returns my affection, whatever be the work she does. If that is the only reason you have for refusing me, I decline to listen to it."

"But there are other reasons," she said, and stopped abruptly.

"Tell them to me, and I will explain them away."

"Oh! you cannot."

"Let me try. I think I have a right to know why you refuse me."

Marion looked around with a troubled face, as if seeking inspiration from earth and sky, before she answered, in a manner that showed how much she doubted the validity of her objection,—"Your position —people would say you derogated from it in marrying a hospital nurse—and not what people term a lady-nurse, but one who has to earn her bread by her work."

Dr. Brandreth laughed, thinking his suit was nearly won. "That's the humility of pride," he said. "But since I must reply to your argument, let me say that I have no position but what I have made for myself, and could be in none that would be too high for you, my queen of women; and for the rest, if you are not a lady because you work for your living, I can be no gentleman, since I work for mine, and therefore we shall be well-matched. So, dear, say the word that will make me happy."

But Nurse Marion only looked more distressed than ever, and again protested, in a sad, faltering voice, "I cannot; it is impossible."

The Doctor's smile passed away. "Marion," he said, "if you have some other reason for rejecting me than the frivolous ones you have given, tell it me and let me combat it."

She was silent for a moment; then she said, brokenly, "I know I should have told you the whole truth at once; but it is still so difficult, after all these years, to speak of that time. There was—once—some one else."

"Dear," said the Doctor, very gently, "I could not imagine that you have come to your present age without winning the love of more than one man; I could scarcely hope that none of them had touched your heart in return. But this some one else,—do you love him still?"

"I can hardly tell," she answered; "it is long ago—more than ten years, and we parted in anger. I had duties which prevented, or which, at least, I thought should prevent my marrying him at once, as he desired. He expected me to consider his wishes before anything else, and quarrelled with me because I could not feel justified in yielding to them."

"And has his anger lasted all these years?"

"I cannot tell. He was going abroad; that was why he was so anxious for an immediate marriage. He sailed for Calcutta within a few weeks of that last interview of ours, and—and—he met a lady on board ship, and married her soon after he reached India. Perhaps he loved her. I hope he did; but sometimes I have fancied—it was vanity on my part, perhaps—that it was only impatience and irritation against me that made him act as he did."

"But, Marion, surely you do not mean to waste your whole life for the sake of a man like that. Do you care for him still?"

"I do not know; but it has never seemed possible for me to think of anyone else."

"You are cherishing a dream. You could give me happiness, and I would devote my life to securing yours. Think twice before you put away my love for the memory of some one, whom not even a woman's imagination can make a hero of. I am not so impatient as that former lover of yours; and I ask you to reconsider your refusal of me. Take a week—no, a month—to think over it. I am not so young that I can afford to wait long for happiness; but I am old enough to know that it may be gained by patience. I shall not in any way try to influence your final decision. Till the month is past our relations shall be those of doctor and nurse, as they have hitherto been. What they shall be afterwards depends on you; but I promise, that whether your answer be 'no' or 'yes,' I will be your friend all my life, and neither fickleness nor petulance shall make me unfaithful to the woman I love. Will you consent to postpone your reply?"

And Marion, glad to put aside at any price a question that aroused such painful memories, promised to yield so far to his wishes.

"I have only to put the whole thing out of my mind," she said to herself, "and a month hence repeat my refusal. Fortunately, I have too much to do to permit of my wasting much time and thought on matters of sentiment."

Marion had indeed plenty of work to do, for she was not one of those nurses who leave the heavier part of the burden to inexperienced probationers. She was too conscious of her duty, both to her patients and her subordinates, to overtax the one or neglect the other. Her care and supervision were no merely nominal things, and perfect content reigned within the bosoms of all whose work lay within the four walls of her ward,—except, perhaps, that of some youthful house-surgeon, whose rash fondness for experiment her experience rebuked. Yet, in spite of all her duties, Dr. Brandreth's proposal would recur to her mind, and as she repeated to herself her one true reason for not accepting it, memories of old days returned to her with a pain whose sharpness seemed in no degree lessened by time.

Ten years is a long time to cherish the memory of a faithless lover, and more than that had elapsed since Jack Holtayne had left her with the bitter, unjust words, "You do not care for me. If you did you would not let me go to India alone."

"I cannot leave my father," she had answered then; and as she reviewed the past, "I could not have left my father," she said to herself now.

(*To be continued.*)

